# Membrane-Active Epithelial Keratin 6A Fragments (KAMPs) Are Unique Human Antimicrobial Peptides with a Non-αβ Structure

**DOI:** 10.3389/fmicb.2016.01799

**Published:** 2016-11-11

**Authors:** Judy T. Y. Lee, Guangshun Wang, Yu Tong Tam, Connie Tam

**Affiliations:** ^1^Department of Ophthalmic Research, Cole Eye Institute and Lerner Research Institute, Cleveland ClinicCleveland, OH, USA; ^2^Department of Pathology and Microbiology, College of Medicine, University of Nebraska Medical CenterOmaha, NE, USA; ^3^Pharmaceutical Sciences Division, School of Pharmacy, University of Wisconsin-MadisonMadison, WI, USA; ^4^Department of Ophthalmology, Cleveland Clinic Lerner College of Medicine of Case Western Reserve UniversityCleveland, OH, USA

**Keywords:** antimicrobial peptides, keratins, epithelial cells, peptide structure and function, innate immunity

## Abstract

Antibiotic resistance is a pressing global health problem that threatens millions of lives each year. Natural antimicrobial peptides and their synthetic derivatives, including peptoids and peptidomimetics, are promising candidates as novel antibiotics. Recently, the C-terminal glycine-rich fragments of human epithelial keratin 6A were found to have bactericidal and cytoprotective activities. Here, we used an improved 2-dimensional NMR method coupled with a new protocol for structural refinement by low temperature simulated annealing to characterize the solution structure of these kerain-derived antimicrobial peptides (KAMPs). Two specific KAMPs in complex with membrane mimicking sodium dodecyl sulfate (SDS) micelles displayed amphipathic conformations with only local bends and turns, and a central 10-residue glycine-rich hydrophobic strip that is central to bactericidal activity. To our knowledge, this is the first report of non-αβ structure for human antimicrobial peptides. Direct observation of *Staphylococcus aureus* and *Pseudomonas aeruginosa* by scanning and transmission electron microscopy showed that KAMPs deformed bacterial cell envelopes and induced pore formation. Notably, in competitive binding experiments, KAMPs demonstrated binding affinities to LPS and LTA that did not correlate with their bactericidal activities, suggesting peptide-LPS and peptide-LTA interactions are less important in their mechanisms of action. Moreover, immunoprecipitation of KAMPs-bacterial factor complexes indicated that membrane surface lipoprotein SlyB and intracellular machineries NQR sodium pump and ribosomes are potential molecular targets for the peptides. Results of this study improve our understanding of the bactericidal function of epithelial cytokeratin fragments, and highlight an unexplored class of human antimicrobial peptides, which may serve as non-αβ peptide scaffolds for the design of novel peptide-based antibiotics.

## Introduction

Antimicrobial resistance is a major public health problem posing deadly threats to the global community. Bacterial infections caused by common pathogens, such as *Escherichia coli, Pseudomonas aeruginosa, Acinetobacter baumannii, Staphylococcus aureus*, and *Streptococcus pneumoniae*, are becoming extremely difficult to treat with current antibiotics (Thabit et al., 2015). Each year hundreds of thousands of people around the world die from infections caused by multidrug-resistant organisms (O'Neill, 2016). Novel classes of anti-infective therapeutics are urgently needed to curtail further deterioration of the situation.

Our skin and mucosal surfaces are under constant challenge by pathogenic microbes in the environment. Production of antimicrobial peptides (AMPs) by epithelial cells and local phagocytes provides a crucial innate defense mechanism guarding the host barriers against microbial invasion. Indeed they are produced by virtually all organisms from bacteria to vertebrates and are best known for their direct and fast-acting microbicidal activity (http://aps.unmc.edu/AP) (Wang et al., 2016). These host defense peptides, primarily defensins and cathelicidins, are short (12-50 amino acids), polycationic, amphipathic peptides possessing α-helical and/or β-sheet structures critical for their antimicrobial activities (Wang, 2014). Furthermore, accumulating evidence has shown the multifaceted contributions of AMPs beyond direct killing of microbes, including modulation of inflammatory responses (Zhang and Gallo, 2016) and promotion of epithelial migration and angiogenesis during wound healing (Mangoni et al., 2016). With advanced technologies in synthesis, functional design and modification of synthetic analogs, cationic AMPs and their modified derivatives are being recognized as promising anti-infective candidates, including six compounds currently in phase I-III clinical trials (Fox, 2013).

Keratins are a large family of intermediate filament proteins comprising the epithelial cell cytoskeleton. Obligate heterodimers and -tetramers of type I keratins (K9-K10, K12-K28, K31-K40) and type II keratins (K1-K8, K71-86) in the cytosol assemble and form a dynamic filament network that remodels upon mechanical, metabolic, and oxidative stresses to preserve the integrity of cell structure and function. Recently, a series of overlapping fragments derived from the C-terminal region (a.a. 515-559) of human K6A were found to be bactericidal (Tam et al., 2012). K6A is a type II keratin constitutively expressed in many types of stratified epithelial cells (Moll et al., 1982) and is upregulated in response to cytokines during inflammation and tissue injury (Coulombe et al., 2004). These glycine-rich human K6A-derived antimicrobial peptides (KAMPs, previously known as KDAMPs) are not constrained by their modest net charge (0 to +2), low helical content (determined by circular dichroism (CD) spectroscopy), or the high salt concentration of the environment, unlike canonical antimicrobial peptides (Tam et al., 2012). In addition, KAMP treatment effectively protects cultured human corneal epithelial cells from bacteria-induced cytotoxicity and bacterial invasion without impacting the morphology and viability of the epithelial cells (Tam et al., 2012).

Here we investigated four KAMP members (Table 1), one of which (KAMP-10) has a net charge of zero (no basic amino acids Lys and Arg) to better understand the structural and mechanistic features of KAMPs. An improved solution nuclear magnetic resonance (NMR) spectroscopy method coupled with low temperature simulated annealing was used to obtain high-quality 3D structure of KAMPs in SDS micelles. While anionic detergent and lipid micelles are both essential mimetic systems of bacterial membranes in solution NMR to obtain high-resolution spectra for structure determination, it has been demonstrated that the 3D structures of human cathelicidin LL-37 and its fragments in complexes with SDS micelles can be applied to their phosphatidylglycerol (PG)- and lipopolysaccharides (LPS)-bound states (Wang, 2008; Wang et al., 2012). In this study, we showed that SDS micelle-bound KAMPs are largely extended without typical α-helix or β-sheet structures, consistent with the structure in the presence of large unilamellar liposomes determined by CD spectroscopy. Mechanistically, peptide binding to the two major bacterial membrane components, LPS and lipoteichoic acid (LTA), is not key to the bactericidal function of KAMPs. Both scanning electron microscopy (SEM) and transmission electron microscopy (TEM) showed dose-dependent disruptive effects of KAMPs on bacterial cell envelopes. While other mechanisms might also be involved, we used co-immunoprecipitation to identify SlyB lipoprotein as a potential bacterial surface ligand during peptide-membrane interactions, and NqrA and ribosomes as potential intracellular targets. Given that the majority of characterized AMPs, as well as all human AMPs to date belong to cationic α, β, or αβ classes (Wang, 2014), this study identifies the first human AMPs with a non-αβ structure, and contributes to the knowledge of unconventional AMPs, especially those that do not rely on defined α-helical or β-sheet structures to facilitate interactions with anionic bacterial membranes and subsequent membrane perturbation.

**Table 1:** **Amino acid sequences of the KAMPs used in this study**.

**Peptide**	**hK6A (NP_005545) a.a**.	**Sequence**	**Net Charge (pH 7.0)**	**Pho%**	**Boman index (kcal/mol)**
KAMP-10	537–546	GGLSSVGGGS	0	20	−0.34
KAMP-18C	533–550	RAIGGGLSSVGGGSSTIK	+2	27	0.57
KAMP-19	533–551	RAIGGGLSSVGGGSSTIKY	+2	26	0.55
KAMP-36	517–552	YGSGLGVGGGFSSSSGRAIGGGLSSVGGGSSTIKYT	+2	22	0.32

*Percentage of hydrophobic amino acids (Pho%), net charge and Boman index (estimated protein-binding potential) of each peptide are calculated using the APD website (Wang et al., 2016). KAMP-18C and KAMP-19 have similar Boman indexes, while shorter and longer peptides have reduced protein binding potential*.

## Materials and methods

### Bacterial strains

*Pseudomonas aeruginosa* corneal isolate 6206 provided by Suzanne M. J. Fleiszig (School of Optometry, University of California, Berkeley, USA), and *S. aureus* 29213 from ATCC were used in this study.

### Peptide synthesis

All peptides used in this study were synthesized by American Peptide Company (now Bachem Americas, Torrance, CA) at >95% purity. Their sequences and net charges were shown in Table 1. Peptide content, purity and sequence were confirmed by amino acid analysis, HPLC and electrospray mass spectrometry. Stock solutions were prepared in sterile distilled water and stored at −20°C. Aliquots were limited to one thaw prior to use.

### Preparation of liposomes

Lipids 1-palmitoyl-2-oleoyl-*sn*-glycero-3-phosphoethanolamine (POPE), 1-palmitoyl-2-oleoyl-*sn*-glycero-3-phospho-(1′-rac-glycerol) (POPG), and 1,2-dielaidoyl-sn-glycero-3-phosphoethanolamine (DEPE) were purchased from Avanti Polar Lipids in chloroform solution. Gel filtration-purified *P. aeruginosa* LPS was purchased from Sigma-Aldrich and reconstituted in distilled water. The large unilamella liposomes were prepared by the freeze-thaw and extrusion method as previously described (Ouberai et al., 2011). Briefly, lipids (1 mg total) in chloroform were evaporated under a dry nitrogen stream to yield a lipid film, then hydrated and vigorously agitated for 2 h with 1 ml of pre-warmed 0.9% saline at 35°C for POPE/POPG liposomes, or with 1 ml of pre-warmed 0.9% saline containing 600 μg *P. aeruginosa* LPS at 45°C for LPS/DEPE liposomes. The liposomal suspensions were subjected to 6 freeze-thaw cycles and 10 passes of extrusion through two stacked polycarbonate membranes with a pore size of 100 nm (Whatman Nuclepore) at 35°C for POPE/POPG liposomes or at 45°C for LPS/DEPE liposomes. The size and monodispersity of liposomes were confirmed by dynamic light scattering with a Zetasizer Nano-ZS (Malvern Instruments). The liposome concentration, represented by total phosphorous content, was quantified by the Barlett assay (Torchilin and Weissig, 2003).

### Particle size and surface charge characterization

Zetasizer Nano-ZS (Malvern Instruments) with an incident beam of He-Ne ion laser (λ = 633 nm) and a 173° detection angle was used to measure particle size dimensions and zeta potentials of peptides, SDS micelles, liposomes, or peptide-associated SDS micelles/liposomes. For the peptide-SDS mixtures, SDS concentration was fixed at 10 mM in water, while peptide concentrations were varied from 21 to 167 μM to achieve 1:480, 1:240, 1:120, and 1:60 peptide-to-SDS molar ratios. For the peptide-liposome mixtures, lipid concentration was fixed at 30 μM in saline, while peptide concentrations were varied from 3.75 to 240 μM to achieve 1:8, 1:4, 1:1, 4:1, or 8:1 peptide-to-lipid molar ratios. Each sample had a volume of 700 μl and was transferred into a low volume disposable sizing cuvette for particle size measurement or a folded capillary cell for zeta potential measurement. The samples were characterized immediately after preparation at 25°C. Each measurement was made in triplicate. Cumulant method and the Stokes-Einstein equation were used to calculate the hydrodynamic diameters (volume average) of particles. The Smoluchowski equation was applied to electrophoretic mobility data to determine zeta potentials.

### CD spectroscopy

CD spectra were acquired at 25°C in a 1.0-mm path-length quartz cell with a Jasco J-815 CD spectropolarimeter. Peptide stocks were diluted in distilled water added to solutions of SDS (2–10 mM in distilled water) or liposomes (0.25–1 mM in 0.9% saline) to obtain a final peptide concentration of 0.1 mM. Each sample was measured immediately after preparation or 24 h post-incubation at 4°C. All samples were scanned from 260 to 195 nm at an interval of 0.1 nm. Spectra were background-subtracted and converted to mean residue molar ellipticity.

### NMR spectroscopy

NMR spectra were acquired at 298K with a Bruker Biospin Avance II 900 MHz spectrometer equipped with a TCI cryoprobe at the California Institute for Quantitative Biomedical Research (QB3), University of California, Berkeley. NMR samples containing 3.4 mM KAMP-10 or KAMP-19 in 0.2 M deuterated SDS (Sigma-Aldrich), 1 mM DSS and 10% D_2_O with an unadjusted pH of 4.5 were prepared and placed in 5 mm NMR tubes (Norell). Two-dimensional DQF-COSY, TOCSY, NOESY and HSQC-TOCSY experiments were conducted and processed with the TopSpin package (Bruker Biospin) for backbone assignment. The mixing times for TOCSY and NOESY experiments were set to 100 ms and 500 ms respectively. Chemical shifts were referenced against DSS. Secondary structural elements were determined by Hα secondary shift analysis. In addition, another set of NMR spectra, including heteronuclear ^15^N and ^13^C 2D correlated spectra under natural abundance, were collected on a 600 MHz Varian NMR spectrometer, processed and analyzed as described previously (Wang, 2008).

### Structural calculations

For structural determination, distance restraints were obtained from two-dimensional NOESY spectra (mixing time 150 ms). The cross peaks were integrated by PIPP (Garrett et al., 1991) and converted to distance restraints 1.8–2.8, 1.8–3.8, 1.8–5.0, and 1.8–6.0 Å corresponding to strong, medium, weak, and very weak types of NOE peaks, respectively. Based on ^1^Hα, ^15^N, ^13^Cα, and ^13^Cβ chemical shifts, backbone angles of micelle-bound peptide were predicted by using an updated version of TALOS (Shen et al., 2009). An extended covalent structure was used as starting coordinates. An ensemble of structures was calculated by using the simulated annealing protocol in the Xplor-NIH program (Schwieters et al., 2003). In all the structures, the polypeptide chain was largely extended with local packing only. In the second round of calculations, a low temperature simulated annealing was conducted at an initial temperature 200 K and high temperature 500 K with 500 cooling steps to generate a structural ensemble that satisfies all the NMR restraints. Structures were accepted based on the following criteria: no NOE-derived distance violations greater than 0.30 Å, backbone dihedral angle violations less than 3°, root mean standard deviations (r.m.s.d.) for bond deviations from ideality less than 0.01 Å, and r.m.s.d. for angle deviations from ideality less than 5°. The structures were viewed and analyzed using PROCHECK (Laskowski et al., 1996) and MOLMOL (Koradi et al., 1996). Structural data were deposited in the Protein Data Bank (PDB ID 5KI0).

### Scanning electron microscopy

Log-phase *P. aeruginosa* or *S. aureus* [200 μl of 10^8^ CFU/ml in saline supplemented with 1% Keratinocyte Basal Medium (Lonza)] were incubated with 0, 45 and 60 μg/ml of KAMP-10 or 0, 60 150 μg/ml of KAMP-18C respectively at 37°C for 3 h. Bacteria were pelleted at 5000 g for 5 min, resuspended in 2.5% glutaraldehyde in PBS and deposited onto a 0.4 μm-pore-size polycarbonate membrane (Whatman Nuclepore) for 1 h at room temperature then 4°C overnight, then post-fixed with 1% osmium tetroxide (OsO_4_) for 1 h, 1% thiocarbohydrazide (TCH) for 5 min, and 1% OsO_4_ again for 5 min (Willingham and Rutherford, 1984). After post-fixation, samples were dehydrated with graded ethanol series, then impregnated with 50% hexamethyldisilazane (HMDS) in ethanol, and finally 100% HMDS. The air-dried samples were sputtered with gold before imaging. SEM images were taken with FEI Helios Nanolab 650 at the Swagelok Center for Surface Analysis of Materials (SCSAM) at Case Western Reserve University.

### Transmission electron microscopy (TEM)

Log-phase bacteria (10^8^ CFU/ml; 1 ml) were treated with KAMPs for 3 h as described in the SEM section. Treated bacteria were pelleted, then fixed with triple aldehyde-DMSO (Fujioka et al., 2012) for 30 min at room temperature. The bacteria were then rinsed with 0.1 M HEPES buffer (pH 7.3) and post-fixed with ferrocyanide-reduced OsO_4_ before embedding. Thin sections were sequentially stained with acidified 2% uranyl acetate followed by a modification of Sato's triple lead stain (Hanaichi et al., 1986). TEM images were taken at the Electron Microscopy Core Facility, School of Medicine, Case Western Reserve University using FEI Tecnai Spirit (T12) with a Gatan US4000 4k × 4k CCD.

### Competitive binding assay

BODIPY-TR-cadaverine (Life Technologies) was prepared in 10 mM NaCl or 5 mM HEPES (pH 7.5) and pre-warmed at 37°C. Purified *P. aeruginosa* LPS and *S. aureus* LTA (Sigma-Aldrich) were prepared in distilled water. Colistin and meropenem (Santa Cruz Biotechnology) were used as positive and negative controls respectively (Ouberai et al., 2011). A final 100 μl mixture of 5 μM BODIPY-TR-cadaverine (in 10 mM NaCl or 5 mM HEPES, pH 7.5), 15 μg/ml LPS or LTA, and peptides or antibiotics in a concentration ranged from 0.02 to 272 μM was added to a 96-well black assay plate. The fluorescence at 580/620 nm was measured at 37°C with a Synergy 4 fluorescence microplate reader (BioTek). Displacement percentage of KAMP relative to colistin and meropenem was determined by: fluorescence of (probe/endotoxin/KAMP mixture - probe/endotoxin/meropenem mixture) / fluorescence of (probe/endotoxin/colistin mixture - probe/endotoxin/meropenem mixture) × 100%.

### Antibacterial activity assay

Lawn culture of *P. aeruginosa* was grown on tryptic soy agar plates at 37°C for 16 h. Starting inoculum at a concentration of 10^8^ CFU/ml was prepared by suspending the bacteria in serum-free Keratinocyte Basal Medium (Lonza) until OD_650_ reading was approximately 0.1. The starting inoculum was diluted 100-fold with 10 mM NaCl or 5 mM HEPES (pH 7.5) resulting 10^6^ CFU/ml bacteria. Stock solution of peptides (or equivalent volume of distilled water as no peptide control) was then added to 10^6^ CFU/ml bacteria and the final concentration of peptides was 200 μg/ml. The mixtures were incubated at 37°C for 3 h. The experiment was run in triplicate. Serial dilutions of the samples at time 0 and 3 h were plated on tryptic soy agar plates and incubated at 37°C overnight for viable bacterial counts in CFU/ml. The percentage killing was determined by: (bacterial count without peptide - bacterial count with peptide) / bacterial count without peptide × 100%.

### Bacterial fractionation and immunoprecipitation

*P. aeruginosa* was pelleted at 5000 g centrifugation for 20 min, then separated into periplasmic, cytoplasmic, inner membrane and out membrane fractions as described previously (Thein et al., 2010). The fractions were dialyzed against 10 mM NaCl overnight, and pre-cleared with Protein A/G resins for 1 h at 4°C with gentle rotation. Rabbit serum containing anti-KAMP-19 antibody (custom made by New England Peptide) (Tam et al., 2012) was covalently coupled to Protein A/G resin (Santa Cruz Biotechnology) with the cross-linker BS^3^ (Thermo Scientific) according to the manufacturer's instructions. Pre-cleared bacterial fraction (0.15–1 mg protein) was mixed with or without 80 μg of KAMP-19 and incubated for 1 h at 4°C with gentle rotation. All the samples were immunoprecipitated with 35 μl of anti-KAMP-19-coupled resins in a total volume of 400 μl for 2 h at 4°C with gentle rotation. Resins were washed 5 times with 10 mM NaCl, and eluted by heating for 5 min with 40 μl of SDS loading buffer (Biorad) containing no reducing agent. The eluates were supplemented with 62.5 mM of DTT (Amresco) and heated again for 5 min prior to 10% Tris-glycine SDS-PAGE. The gel was treated with silver staining (Thermo Scientific).

### Mass spectrometry

Mass spectrometry was performed at the Lerner Research Institute Proteomics Core at the Cleveland Clinic. Protein bands (within the areas where band patterns of IP and control were different) were cut out from the gel and digested with trypsin for LC-MS analysis. The LC-MS system used was a Finnigan LTQ-Obitrap Elite hybrid mass spectrometer system. The HPLC column was a Dionex 15 cm × 75 μm i.d. Acclaim Pepmap C18, 2 μm, 100 Å reversed phase capillary chromatography column. Five microliters volumes of the extract were injected and the peptides eluted from the column by an acetonitrile/0.1% formic acid gradient at a flow rate of 0.25 μl/min were introduced into the source of the mass spectrometer on-line. The microelectrospray ion source is operated at 2.5 kV. The digest was analyzed using the data dependent multitask capability of the instrument that acquired full scan mass spectra to determine peptide molecular weights and product ion spectra to determine amino acid sequence in successive instrument scans. The data were analyzed by using all CID spectra collected in the experiment to search the *P. aeruginosa* reference sequence database with the programs Mascot and Sequest.

### Statistical analysis

Data are expressed as mean ± SD, calculated from triplicate samples per group in each experiment. Each experiment was performed individually at least three times. Statistical significance of differences between 2 groups was determined by the Student's *t*-test (two-tailed). *P* < 0.05 was considered significant.

## Results

### KAMPs interact with bacterial membrane mimetics

**Figure 1:**
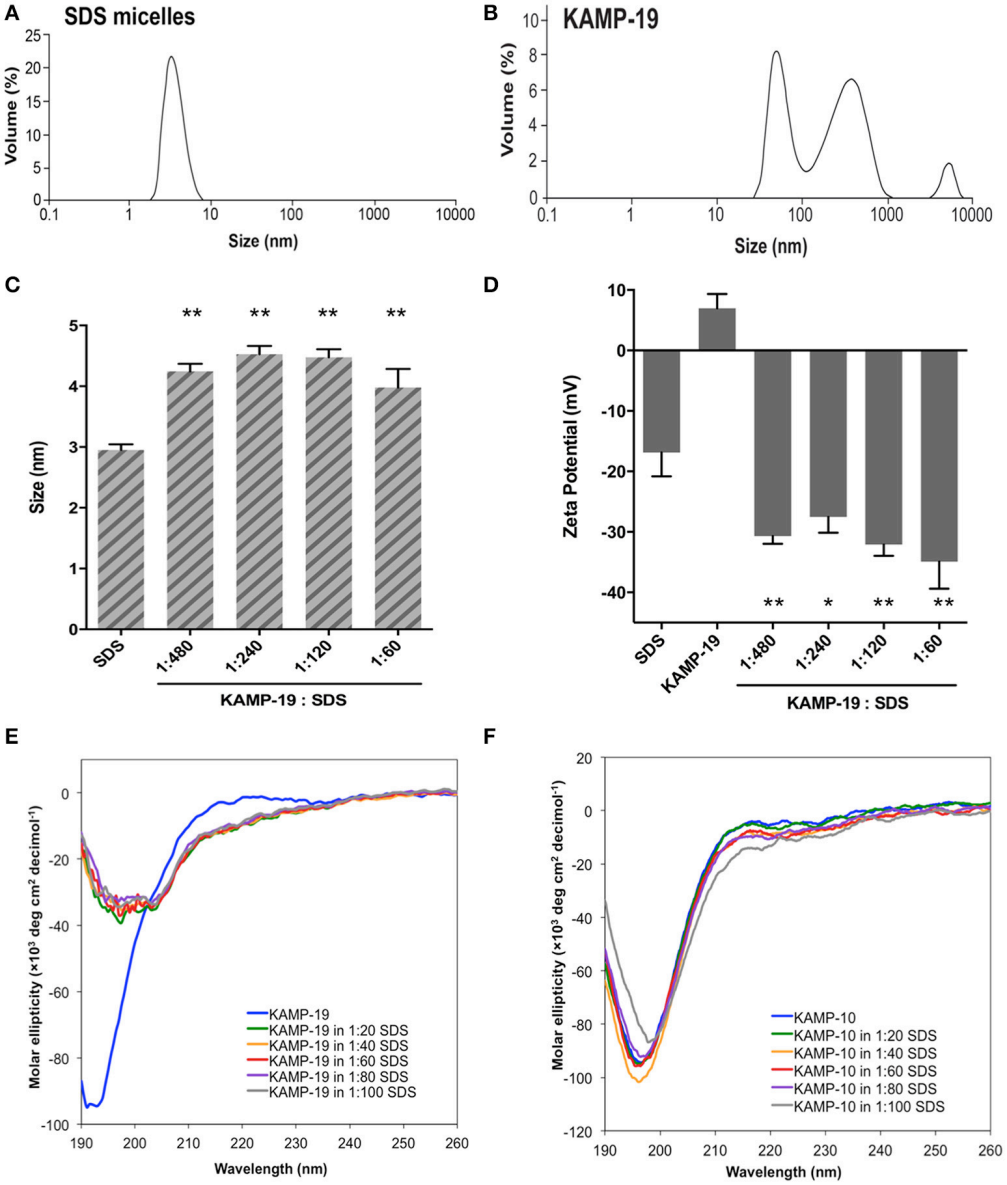
**Volume-based particle size distributions, zeta potentials and circular dichroism spectra of KAMP/SDS micelle complexes. (A)** SDS micelles (10 mM in water) are monomodal in size with hydrodynamic diameter of ~3 nm. **(B)** KAMP-19 (167 μM in water, the concentration used in 1:60 peptide/SDS mixture for **(C)** and **(D)**) showed two major populations of particle size with hydrodynamic diameters of ~50 and ~400 nm. After mixing with KAMP-19, **(C)** the particle sizes and **(D)** the magnitudes of zeta potential of anionic SDS micelles were increased. **(E)** The CD spectrum of KAMP-19 in water showed unstructured random coil conformation. Increasing the concentration of SDS did not change the overall random coil conformation. Similarly, **(F)** KAMP-10 remained random coil-like in the presence of SDS. Increasing the concentration of SDS did not induce helical structure. Mean ± SD is shown. ^*^p < 0.05, ^**^p < 0.005 (*t*-test, two-tailed).

**Figure 2:**
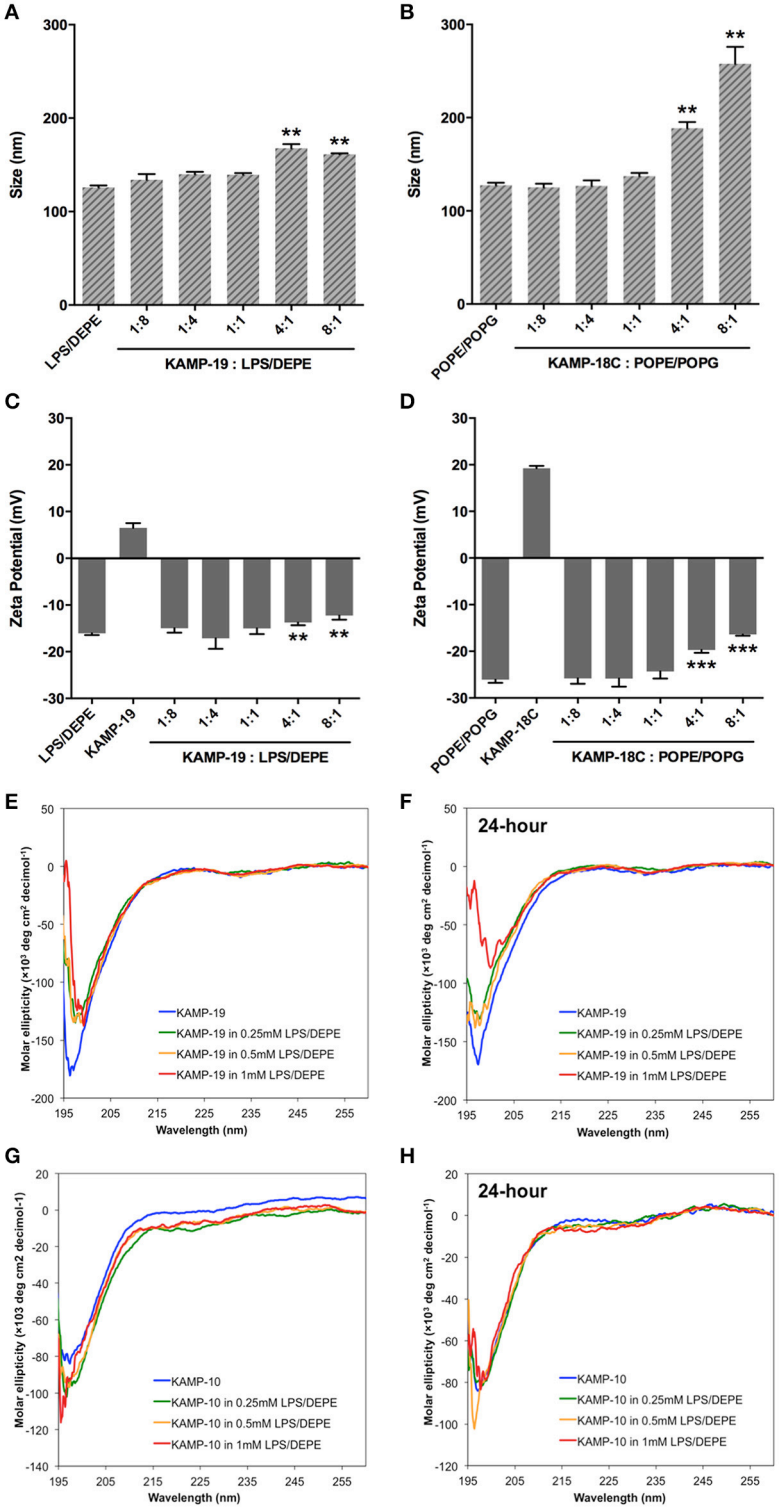
**Volume-based particle size distributions, zeta potentials and circular dichroism spectra of KAMP/large unilamellar liposome complexes. (A)** LPS/DEPE liposomes (30 μM in saline) and **(B)** POPE/POPG liposomes (30 μM in saline) were monomodal in size. After mixing with KAMP-19 or KAMP-18C, the particle size of **(A)** LPS/DEPE liposomes and **(B)** POPE/POPG liposomes increased. Anionic **(C)** LPS/DEPE liposomes and **(D)** POPE/POPG liposomes displayed less negative zeta potential after positively charged KAMP-19 or KAMP-18C was added. **(E)** Immediately after mixing with LPS/DEPE liposomes, KAMP-19 showed decreased magnitude of ellipticity without changing the overall random coil conformation. **(F)** Consistent observation was recorded after KAMP-19 had been incubated with the liposomes for 24 h. Similarly, **(G)** ellipticity of KAMP-10 remained the same immediately after mixing with the liposomes. **(H)** Longer incubation time (24 h) also did not make any difference. Mean ± SD is shown. ^**^p < 0.005, ^***^p < 0.0005 (*t*-test, two-tailed).

SDS micelles, as well as large unilamellar liposomes are commonly used models of membranous environments. The zwitterionic phosphatidylethanolamine (PE) and the anioinc phosphatidylglycerol (PG) are the major components of bacterial plasma membranes (Warschawski et al., 2011). Therefore, POPE/POPG have been used in numerous studies to mimic bacterial cytoplasmic membranes of both Gram types. In addition to PE, anionic LPS is a major structural component of the outer membrane of Gram-negative bacteria. It has been shown that LPS can be incorporated into DEPE membranes without disturbing membrane morphology (Nomura et al., 2011), rendering it a great model of bacterial outer membranes (Su et al., 2011). To conduct an initial investigation of potential interactions between KAMPs and bacterial membranes, we employed the dynamic and electrophoretic light scattering techniques to measure particle size distributions and zeta (ζ) potentials of the membrane mimetics alone and mixed with KAMPs (Domingues and Santos, 2010; Freire et al., 2011; Naskar et al., 2013). As shown in Figures 1A, 2A,B, the average volume hydrodynamic diameters of SDS micelles, LPS/DEPE liposomes and POPE/POPG liposomes were 2.95 ± 0.09, 126.0 ± 2.1 and 127.5 ± 2.7 nm respectively, indicating monomodal size distribution with low polydispersity. High concentration of KAMP-19 alone (167 μM) had two major particle size populations with average volume hydrodynamic diameters of ~50 and ~400 nm (Figure 1B). It has been reported that glycine and serine residues in transmembrane helices may facilitate self-oligomerization (Smith et al., 2013). Upon addition of KAMP-19 at various molar ratios (including the molar ratio used in the subsequent 3D structural characterization by NMR spectroscopy), hydrodynamic diameters of SDS micelles increased (*p* < 0.005) (Figure 1C). Peptide-liposome complexes also increased in size with increasing peptide-to-lipid (P/L) molar ratios (Figures 2A,B). Specifically, at 8:1 (P/L) ratio, the average volume hydrodynamic diameters of KAMP-19/LPS/DEPE and KAMP-18C/POPE/POPG liposome complexes were found to be 161.4 ± 1.0 and 257.9 ± 18.2 nm respectively (*p* < 0.005). With regard to the peptide effects on surface potential of membrane mimetics, the magnitude of zeta potential for the anionic SDS micelles was increased significantly (*p* < 0.01) in the presence of KAMP-19 (Figure 1D), suggesting that DS^−^ monomers in the aqueous medium were drawn toward the micelles by the positively charged KAMP-19 at the interface. It has been reported that the hydrophobic portions of classic amphipathic helices penetrate into the interior of SDS or dioctanoylphosphatidylglycerol (D8PG) micelle cores, while the cationic amino acids such as arginines directly interact with the anionic headgroups (Wang et al., 1996; Wang, 2008). In agreement with literature (Den Hertog et al., 2004; Ringstad et al., 2006; Manzini et al., 2014), zeta potentials of anionic LPS/DEPE liposomes (−16.07 ± 0.35 mV) and POPE/POPG liposomes (−26.10 ± 0.66 mV) became less negative when they were mixed with increasing concentration of cationic KAMP-19 and KAMP-18C respectively (Figures 2C,D), indicating surface charge neutralization by KAMPs. The change of liposome zeta potentials became significant when the molar ratios of peptide to lipid were higher than 1:1. Specifically, at 4:1 and 8:1 (peptide/lipid) molar ratios, KAMP-19/LPS/DEPE complexes exhibited zeta potentials of −13.77 ± 0.55 mV and −12.27 ± 0.87 mV respectively (*p* < 0.005), and KAMP-18C/POPE/POPG complexes displayed −19.73 ± 0.59 mV and −16.40 ± 0.26 mV (*p* < 0.0005). Collectively, the data indicated KAMP interactions with both detergent and lipid membrane mimetics and validated their application in the subsequent study of membrane-bound structures of KAMPs.

### CD characterization of peptide conformations in complex with bacterial membrane mimetics

Regular conformations (i.e., α-helix, β-sheet) that are amphipathic are essential for most AMPs to destabilize the bacterial membrane. Previously, CD spectroscopy was used to estimate the secondary structures of various KAMPs, including KAMP-10 and KAMP-19, in aqueous (water) or hydrophobic (2,2,2 trifluoroethanol) media, and no induced helical conformations were found (Tam et al., 2012). Here, to determine the conformations of KAMPs in complex with bacterial cell model membranes, KAMP-19 and KAMP-10 were individually mixed with increasing concentration of SDS micelles or LPS/DEPE liposomes and their CD spectra were analyzed. KAMP-19 alone in distilled water (Figure 1E) or saline (Figure 2E) displayed the characteristic CD spectrum of random-coil as expected, which was indicated by strong negative ellipticity at the 195–200 nm region and near-zero ellipticity above 210 nm. Comparing with the signature CD spectrum of α-helix which displays two negative troughs at 208 nm and 222 nm and a positive peak at 193 nm, the overall conformations of KAMP-19 remained as random coils in the presence of SDS micelles (Figure 1E) or LPS/DEPE liposomes (Figure 2E), albeit the magnitude of ellipticity at the 195–200 nm decreased suggesting a propensity for a more ordered structure. Higher peptide-to-micelle or peptide-to-lipid ratios did not make significant difference to the unstructured conformation of KAMP-19. We also prolonged the incubation time prior measurements to allow ample time for peptide interactions with bacterial membrane mimetics, yet no induction of secondary structure was observed (Figure 2F). A similar analysis was conducted with KAMP-10 and the observations were consistent (Figures 1F, 2G,H). Although a potential conformational change in the case of KAMP-10 is not a clear cut as the case of KAMP-19, these CD spectra indicated that KAMPs in complex with biomembrane-like liposomes and with SDS micelles display similar random coil conformations.

### NMR structures of KAMP-10 and KAMP-19 bound to membrane-mimetic micelles

**Figure 3:**
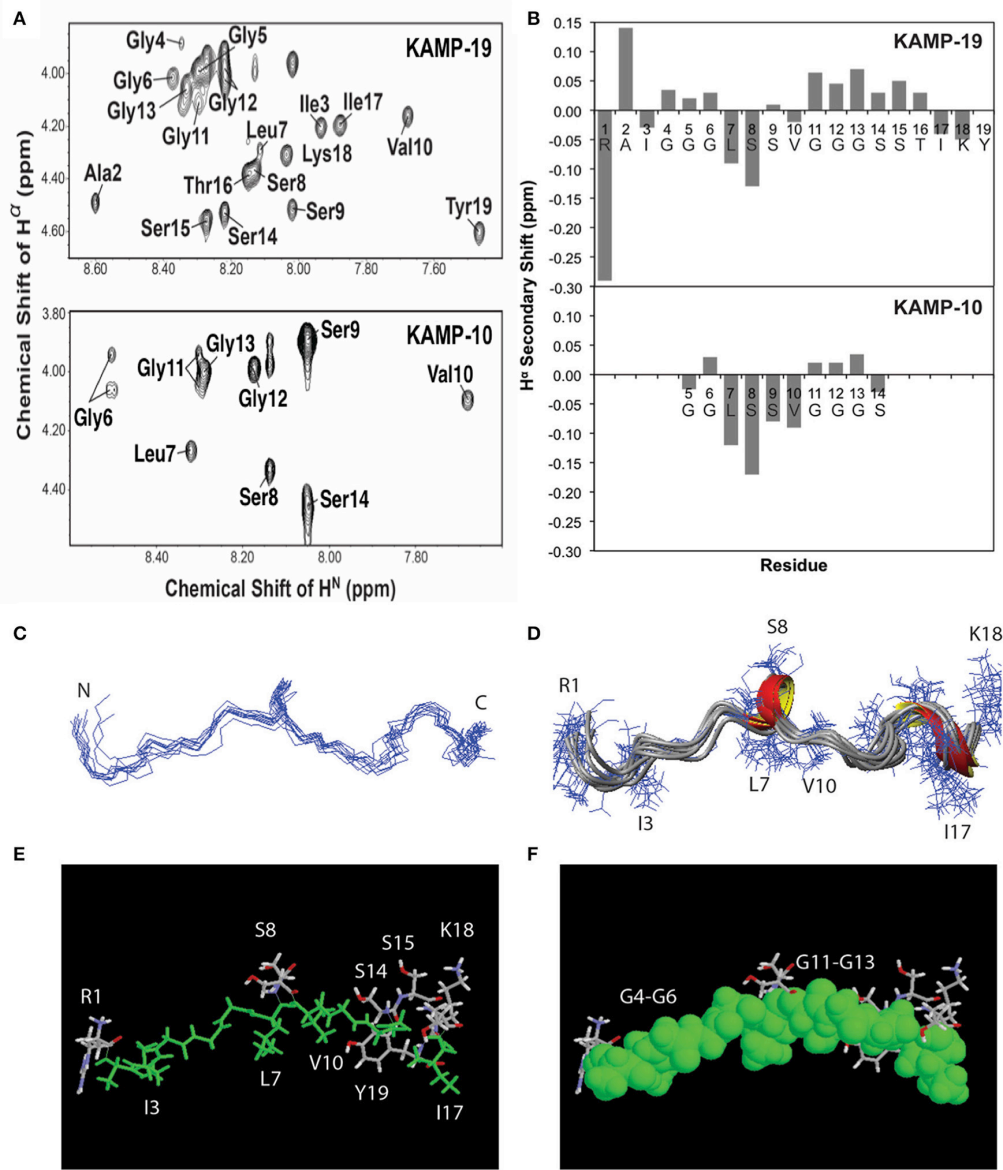
**NMR structures of KAMP-19 and KAMP-10 bound to SDS micelles at a peptide/SDS molar ratio of 1:60, pH 5.4 and 25°C. (A)** Well-resolved NMR spectra of KAMP-19 (upper) and KAMP-10 (lower) indicated low randomness. **(B)** Hα secondary chemical shifts analysis of KAMP-19 (*upper*) and KAMP-10 (*lower*) in SDS micelles. **(C)** NMR structure of KAMP-19 bound to SDS micelles was refined. Superimposed backbone view of 10 structures of KAMP-19. The r.m.s.d. for superimposing residues 2–18 is 0.57 Å. **(D)** Ribbon diagram of KAMP-19 with 3_10_ helical turns between residues 7–9 and 15–17. **(E)** Stick view of the KAMP-19 structure with hydrophobic residues in green. **(F)** The same view as in **(E)** and the hydrophobic residues (*green*) are represented in the space filling model. In both **(E)** and **(F)**, hydrophilic side chains (in *white, red*, and *blue*) are limited to three regions in the view with a majority clustered at the C-terminus (red = oxygen and blue = nitrogen). In addition, the aromatic ring of the C-terminal tyrosine can be seen to pack with the hydrophobic strip.

To determine the structures of KAMPs at the atomic level, NMR signal resonances were assigned using the established method of Wüthrich (Wüthrich, 1986) based on two-dimensional DQF-COSY, TOCSY, and NOESY experiments. As an example, the proton assignments for the fingerprint regions of KAMP-10 and KAMP-19 in complex with SDS micelles are shown in Figure 3A. The secondary Hα chemical shifts of KAMP-19 and KAMP-10 in SDS micelles were calculated based on the random chemical shifts of Wüthrich (Wüthrich, 1986) and presented in Figure 3B. In this plot, a string of negative deviations (due to up field shifts) suggests α-helical conformations, while a string of positive shifts (due to down field shifts) implies β-strands. However, these deviations are relatively small (mostly −0.15 or less) and local compared to those of human cathelicidin LL-37 (mostly −0.2 to −0.7) bound to SDS (Wang, 2008). Thus, a regular helical structure or β-stand is unlikely, consistent with CD spectra (Figures 1E,F). However, we do not exclude a local helical turn spanning residues Leu7-Ser9 in KAMP-19. This helical turn appears to be conserved in KAMP-10 (residues Leu7-Ser9) (Figure 3B). Indeed, structural calculations revealed such a helical turn between residues 7–9 in KAMP-19.

To further refine the atomic structure of KAMP-19 in bacterial membrane-mimetic environments, we used an improved 2D NMR method, which includes the use of natural abundance 2D heteronuclear spectra (Wang et al., 2005). The importance of this improved NMR method in spectral resolution and thus the accuracy of structure determination has been demonstrated recently (Wang, 2013). The NMR restraints used for structural determination include 94 distance restraints and 18 backbone dihedral angle (ψ and Φ) restraints derived from chemical shift analysis. Since there are no long-range NMR restraints and the overall structure is largely extended, we also refined the structure by using low temperature simulated annealing, from which we generated a structural ensemble (Figure 3C). Analysis of this structural ensemble revealed two one-turn 3_10_ helices between residues 7–9 and 15–17, indicating the structure is largely extended with local bends and turns (Figure 3D). This structure is unique because it differs from known structures of human AMPs that consist of α-helix (e.g., LL-37), β-sheet (e.g., α-defensins), or mixed α and β structures (e.g., β-defensins) (Wang, 2014). Furthermore, when the hydrophobic residues are colored in green (including glycines in rasmol analysis), a nearly continuous string of hydrophobic groups is revealed (Figures 3E,F). Interestingly, the hydrophilic side chains are sparse and only appear at the N-terminus (Arg1), center (Ser8 and Ser9), and C-terminus of the peptide chain (Ser-Ser-Thr and Lys-Tyr) (Figures 3E,F). Therefore, the overall structure of KAMP-19 is amphipathic. Such an amphipathic structure sheds light on the molecular basis of membrane binding of KAMP-19. In this structure, the C-terminal aromatic ring of Tyr19 is adjacent to the hydrophobic surface. This tyrosine may be involved in membrane binding. There is precedent for such a role of interfacial tyrosine bound to micelles (Wang et al., 1996). In addition, basic side chains can also interact with anionic membranes, especially when located in the interface of the amphipathic structure (Wang, 2007). However, these hydrophilic residues in KAMP-19 did not appear to be important here because they are dispensable. KAMP-13, obtained after removing the N-terminal hydrophilic Arg1 and C-terminal Ser-Thr-Ile-Lys-Tyr sequence of KAMP-19, is equally active against *P. aeruginosa* (Tam et al., 2012). KAMP-10 is also equally active against *P. aeruginosa*. Since the structure is conserved in shorter peptides such as KAMP-10 (Figure 3B), we propose the central glycine-containing hydrophobic strip of KAMP-19 is critical for membrane binding to inhibit this bacterium. This is consistent with previous findings that glycine-to-alanine mutations (G6A and G12A) in KAMP-10 significantly reduced its bactericidal activity against *P. aeruginosa* (Tam et al., 2012). Such a mode of membrane binding of KAMP peptides is different from canonical amphipathic helices of human LL-37, where cationic side chains can play an important role in membrane permeation (Wang, 2008; Wang et al., 2014).

### KAMPs induce bacterial cell envelope damages in a concentration-dependent manner

KAMP-targeted bacterial cells appeared to have compromised cell membrane integrity, and killing activity of KAMPs was rapid, suggesting direct membrane perturbation might be involved in their mechanism of action (Tam et al., 2012). To characterize the ultrastructural changes in bacterial cell envelopes caused by KAMPs, we used both SEM and TEM to directly observe two clinically important pathogens, Gram-negative *P. aeruginosa* and Gram-positive *S. aureus*, at high density (10^8^ CFU/ml each in saline) and treated with a bacteriostatic dose of KAMP-10 (45 μg/ml) and KAMP-18C (60 μg/ml) respectively. The bacterial inhibition doses were defined as the concentrations at which no bacterial growth was observed. Bacterial killing was verified by the reduction of viable bacterial counts after 3 h of incubation in 150 mM NaCl solution. The choice of KAMPs was based on their activity spectra determined previously (Tam et al., 2012) and the consideration that using those KAMPs with moderate activity toward the two pathogens would allow us to detect subtle changes in membrane integrity.

**Figure 4:**
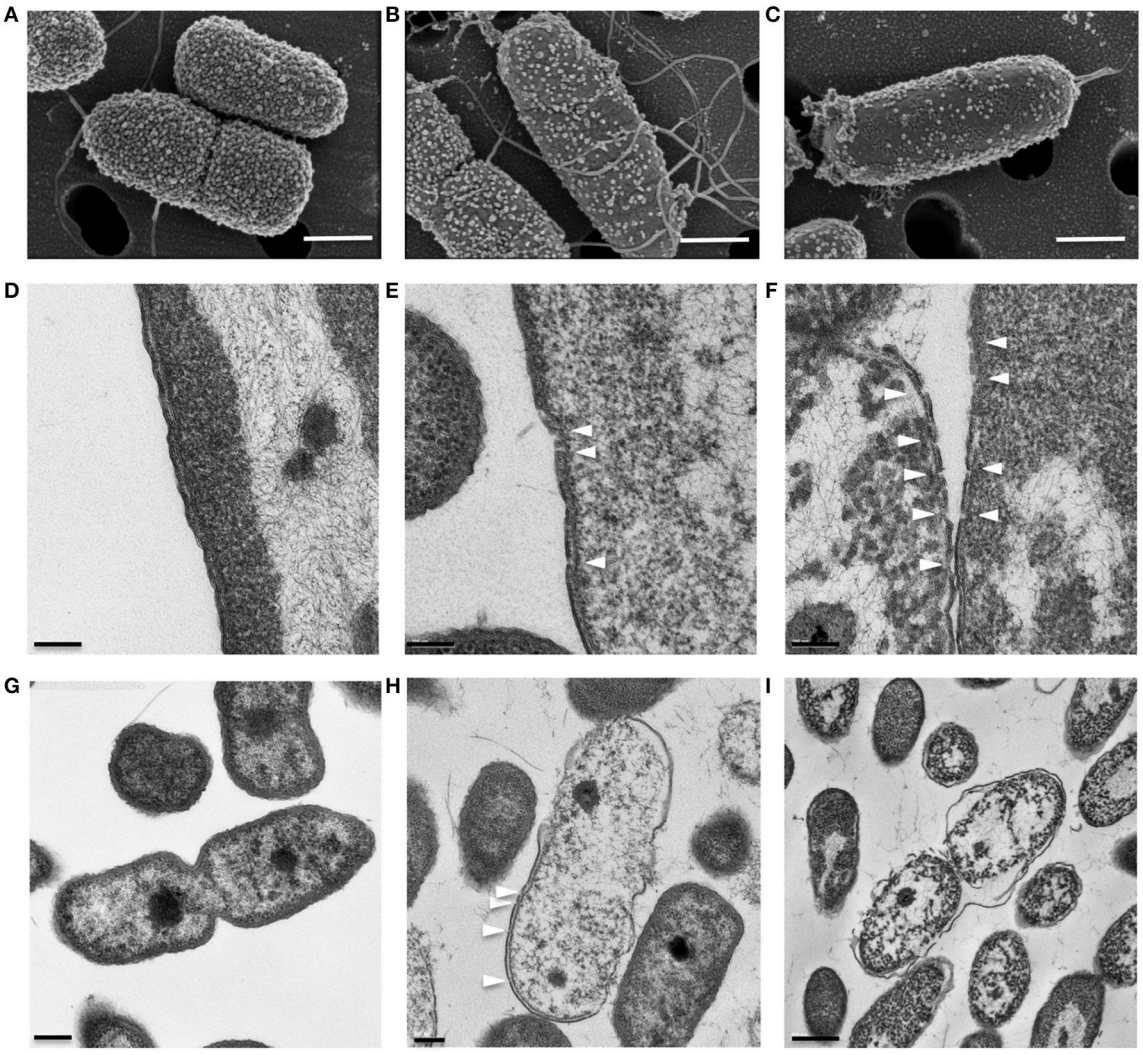
**High-resolution (A–C)** scanning and **(D–I)** transmission electron micrographs of *P. aeruginosa* (10^8^ CFU/ml) treated with KAMP-10 in saline at 37°C for 3 h. **(A,D,G)** Untreated control cells. **(B,E,H)** Cell treated with KAMP-10 at bacteriostatic concentration (45 μg/ml). Loss of bacterial surface vesicle-like components was observed. Pores were mostly found on the bacterial outer membrane (*arrows*). **(C,F,I)** Cells treated with KAMP-10 at bactericidal concentration (60 μg/ml). The cell envelope showed severe damages and peeling. More pores were formed and they penetrated both the outer and cytoplasmic membranes (*arrows*) leading to leakage of cytoplasmic contents. Scale bar, 500 nm **(A–C)**, 100 nm **(D–F)**, 200 nm **(G,H)** or 500 nm **(I)**.

SEM and TEM showed that untreated *P. aeruginosa* control cells were undergoing cell division and possessed densely populated, membrane vesicle-like surface structures (Figure 4A) as well as intact outer and cytoplasmic membranes (Figures 4D,G). In contrast, *P. aeruginosa* treated with a growth inhibitory concentration of KAMP-10 did not undergo cell division and demonstrated partial loss of vesicle-like surface structures (Figure 4B) and formation of pores on the outer membrane (Figures 4E,H). These effects became more severe when the concentration of KAMP-10 was increased to bactericidal (60 μg/ml). Treated *P. aeruginosa* showed substantial loss of surface structures and peeled cell envelope (Figure 4C) in addition to pore (channel-like) formation in outer and cytoplasmic membranes, and in some cases complete membrane disintegration (Figures 4F,I).

**Figure 5:**
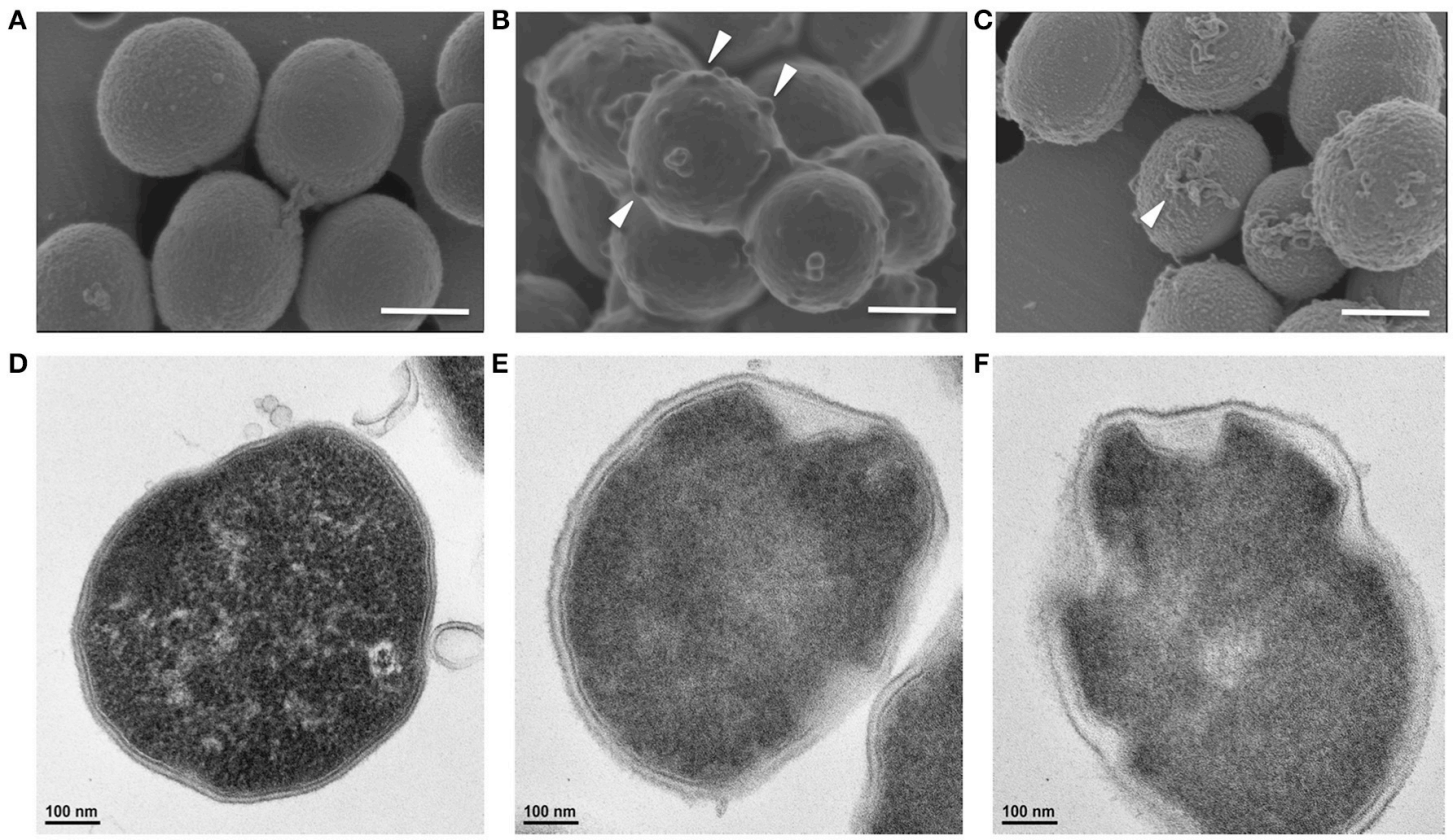
**High-resolution (A–C)** scanning and **(D–F)** transmission electron micrographs of *S. aureus* (10^8^ CFU/ml) treated with KAMP-18C in saline at 37°C for 3 h. **(A,D)** Untreated control cells. **(B,E)** Cells treated with KAMP-18C at bacteriostatic concentration (60 μg/ml). Blisters (*arrows*) began to form on the cell surface. The cytoplasmic membrane retracted from the cell wall. **(C,F)** Cells treated with KAMP-18C at bactericidal concentration (150 μg/ml). Blisters were burst and holes remained on the cell envelope. Spillage of cell contents was observed. More irregular membrane retractions were also found. Scale bar, 500 nm **(A–C)** or 100 nm **(D–F)**.

Similarly, Gram-positive *S. aureus* treated with a growth inhibitory concentration of KAMP-18C showed deformed morphology of the cell envelope (Figure 5B) compared to untreated control cells (Figure 5A). Using SEM, multiple blisters were clearly visible on the surface of each bacterial cell. Most of the blisters appeared to be open when the concentration of KAMP-18C was increased to bactericidal (150 μg/ml) (Figure 5C). In parallel, TEM of untreated *S. aureus* showed intact and discrete cell walls and cytoplasmic membranes (Figure 5D), whereas treated bacteria showed irregular cytoplasmic retraction with cell wall detachment at multiple locations in the cells (Figure 5E). The severity of cell envelope deformation and the appearance of discontinuous cytoplasmic membranes were increasingly notable when KAMP-18C was increased from bacteriostatic to bactericidal concentration (Figure 5F).

### Binding to LPS or LTA is not required for antibacterial action of KAMPs

Many AMPs are reported to bind endotoxins to gain access to bacterial membranes and facilitate subsequent membrane disruption or translocation into bacteria. To determine whether the killing mechanism of KAMPs involves interactions with LPS or LTA, we evaluated the binding affinities of KAMP-10 and KAMP-19 to *P. aeruginosa* LPS and of KAMP-18C and KAMP-36 to *S. aureus* LTA using a competitive binding assay, and correlated the relative binding affinity with the killing activity. This assay used a fluorescent probe, BODIPY-TR-cadaverine (BC), which is quenched when bound to LPS or LTA, and dequenched when competitively displaced and released from the complex, as manifested by the increase of fluorescence detection (Wood et al., 2004). Since binding affinities can be affected by environmental factors, we conducted the assays in two vehicles containing either sodium chloride or HEPES. The former is a salt solution with intrinsic acidity (pH ~5.5), and the latter is a zwitterionic buffer at neutral pH (pH 7.5).

**Figure 6:**
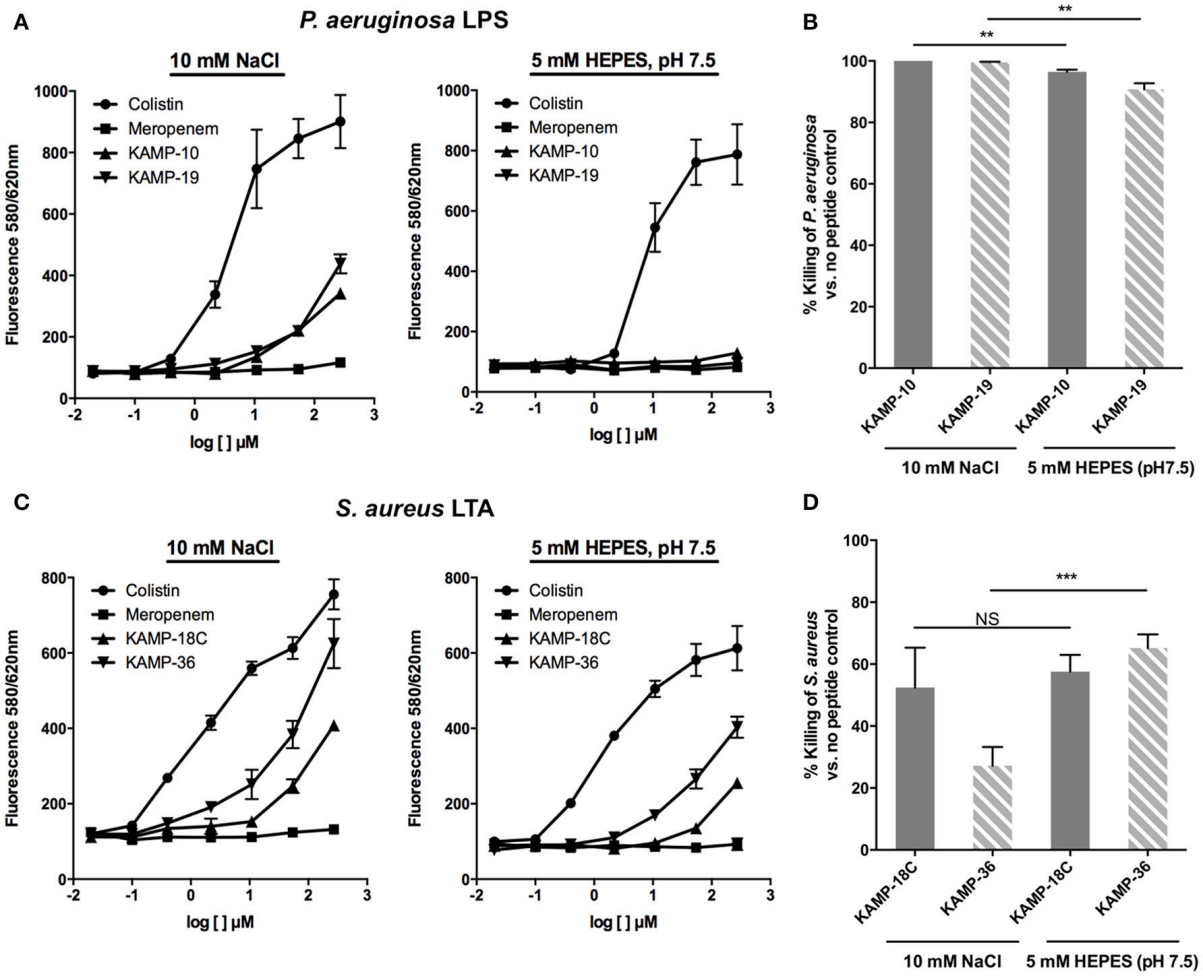
**Binding affinity of KAMPs to ***P. aeruginosa*** LPS or ***S. aureus*** LTA measured by the competitive binding assay**. LPS- or LTA-bound BODIPY TR cadaverine in 10 mM NaCl or 5 mM HEPES (pH 7.5) was competitively displaced by agents having affinity of LPS or LTA, resulting in increased fluorescence signal. **(A)** KAMP-19 and KAMP-10 showed relatively low binding affinities to *P. aeruginosa* LPS in NaCl (*left*) and no binding in HEPES (*right*). **(B)** Using the same media, both KAMPs were highly effective against *P. aeruginosa*. **(C)** KAMP-18C and KAMP-36 displayed lower binding affinities for LTA in HEPES compared with NaCl. However, **(D)** these peptides showed similar (KAMP-18C) or stronger (KAMP-36) bactericidal activities in HEPES against *S. aureus*. Mean ± SD is shown. ^**^p < 0.005, ^***^p < 0.001 (*t*-test, two-tailed).

When compared with colistin, known to bind LPS and LTA and interact with bacterial membranes to exert its killing effect (100% fluorescent probe displacement), and meropenem, an antibiotic that inhibits bacterial cell wall synthesis (0% displacement), KAMP-10 and KAMP-19 showed up to 28.7 and 41% displacement respectively at 270 μM in 10 mM NaCl (*p* < 0.001 vs. colistin), indicative of relatively low affinities of both peptides for *P. aeruginosa* LPS (Figure 6A). Nonetheless, the peptides were highly effective at killing *P. aeruginosa* (near 100% cell death; *p* < 0.0001 vs. no peptide control in each instance) (Figure 6B). When KAMP-10 or KAMP-19 was added to BC:LPS complex in 5 mM HEPES buffer, neither peptide displayed binding affinity for LPS (Figure 6A), however both peptides remained highly active in killing *P. aeruginosa* (96.5 and 90.8% killing respectively, *p* < 0.0001 vs. no peptide control), albeit their activities were reduced (*p* < 0.005) (Figure 6B). The data suggested that interactions with LPS might enhance, but were not required for bactericidal activity of KAMPs. This observation is consistent with a previous report, which showed that the bactericidal activity of KAMP-19 was not reduced by LPS mutation (i.e., incomplete LPS core oligosaccharide and/or O-antigen deficiency) in *P. aeruginosa* (Tam et al., 2012).

In the case of *S. aureus* LTA, as evident from Figure 6C, both KAMP-18C and KAMP-36 showed low to moderate affinities in HEPES buffer (up to 31.3 and 59.7% displacement respectively at 270 μM, *p* < 0.005 vs. colistin). The affinities significantly increased (up to 44.3% and 79.1% displacement respectively) in NaCl solution (*p* < 0.05). While both KAMPs displayed lower LTA binding affinities in HEPES than in NaCl solution, killing activities were found to be similar for KAMP-18C or significantly higher for KAMP-36 (65.3% killing in HEPES vs. 27.2% in saline, *p* < 0.001) (Figure 6D). These data indicated that *S. aureus* killing by KAMPs was not correlated with LTA binding. Similar to anionic capsule polysaccharides produced by some bacteria that limit the amount of antimicrobial peptides reaching their surface (Llobet et al., 2008), LTA might be able to sequester KAMPs and limit their access to the bacterial cell membrane.

### Additional interaction partners of KAMPs

It is known that some AMPs have dual bactericidal mechanisms: membrane disruption and inhibition of intracellular targets (Wang et al., 2015). In addition, the bactericidal activity of KAMPs against *P. aeruginosa* is not affected by high salt concentration (150 mM NaCl), suggesting that KAMPs might directly interact with bacterial surface components. To identify potential KAMP-interacting bacterial proteins, lysate fractions of *P. aeruginosa* enriched with cytoplasmic proteins, periplasmic proteins, outer membrane proteins or inner membrane proteins were incubated with KAMP-19, and immunoprecipitated using anti-KAMP-19 antibody-coupled resin. Mass spectrometry was used to identify the proteins in the immunoprecipitates. The relative quantity of identified proteins was indicated by spectral counts (SC). Proteins with SC ≥ 10 were considered abundant. Proteins that were much more abundant in the KAMP-19-incubated bacterial fractions (IP) compared to the corresponding no-peptide controls (i.e., those specifically pulled down by KAMP-19) were considered as those with IP/control SC ratio ≥ 10. Using this method, we identified 9 abundant proteins (SC ≥ 10) specifically bound to KAMP-19 (IP/control SC ratio ≥ 10) (Table 2), including one outer membrane protein (hypothetical protein PSPA7_4325), which is 99% identical to the outer membrane lipoprotein SlyB of *P. aeruginosa* strain PAO1, and one cytoplasmic membrane associated protein - sodium-translocating NADH-quinone oxidoreductase (Na^+^-NQR) subunit A (NqrA). The remaining seven are intracellular proteins - recombinase A (RecA), ATP-dependent RNA helicase (RhlE), ribosomal large subunit pseudouridine synthase B and C (RluB and RluC), 30S ribosomal proteins S2 and S7, and 50S ribosomal protein L14; all except RecA are involved in normal ribosome assembly, stability and efficient protein synthesis.

**Table 2:** **Mass spectrometry identified ***P. aeruginosa*** proteins co-immunoprecipitated with KAMP-19**.

**NCBI Accession**	**Protein**	**Category**	**Molecular weight (kDa)**	**SC Counts**		**SC ratio (IP/control)**
				**IP**	**Control**	
WP_003092260	Recombinase A (RecA)	DNA repair	37	16	1	16.0
WP_017001581	ATP-dependent RNA helicase (RhlE)	Ribosome assembly	70	10	1	10.0
WP_012075012	Hypothetical protein PSPA7_1951 (ortholog of Ribosomal large subunit pseudouridine synthase B, RluB)	Ribosome assembly	44	16	0	IP only
WP_012075168	Ribosomal large subunit pseudouridine synthase C (RluC)	Ribosome assembly	36	13	1	13.0
WP_003092394	30S ribosomal protein S2	Ribosome assembly	27	18	1	18.0
WP_003093742	30S ribosomal protein S7	Ribosome assembly	17	14	1	14.0
WP_003093714	50S ribosomal protein L14	Ribosome assembly	13	10	0	IP only
WP_003109479	Na^+^-translocating NADH-quinone oxidoreductase subunit A (NqrA)	Ion pump	48	11	1	11.0
WP_012076740	Hypothetical protein PSPA7_4325 (ortholog of SlyB)	Outer membrane lipoprotein	16	10	1	10.0

*Proteins with both Spectral Count (SC) and Spectral Count Ratio (IP:control) ≥ 10 are shown*.

## Discussion

Keratins are essential components of the epithelial cell cytoskeleton. Previously, we reported that bactericidal fragments of keratin 6A (KAMPs) have neutral or modest positive charge and are expressed in stratified epithelial cells, suggesting a novel, direct antimicrobial function for keratins in epithelial innate defense against infection. The current study characterized the 3D structure of KAMPs and explored their mode of action at the bacterial membrane interface. We show that micelle-bound KAMPs do not display conventional α-helical or β-sheet structure. While they induce ultrastructural damage to the bacterial cell envelopes, LPS or LTA binding is not involved to facilitate their bactericidal activity. We further identified a hypothetical lipoprotein (PSPA7_4325) of *P. aeruginosa* as a potential outer membrane target of KAMPs, and several other proteins, including NqrA, a cytoplasmic membrane-bound respiratory enzyme subunit, and the ribosome machine as potential intracellular targets of KAMPs. These data shed light on the bactericidal mechanism of this distinct family of epithelial-derived AMPs.

AMPs are diverse, but can be classified into four unified classes based on the types of secondary structures (Wang, 2010). The first class is α-helical peptides; the second class consists of β-sheet structures. While the αβ class contains both α and β structures, the non-αβ class has neither α nor β structure. Indeed, the majority of AMPs possess positive net charges and strong helical and/or beta structures; anionic or neutral peptides or those with low propensity to form defined secondary structures are rarely reported. In humans, there are only three reported classes of AMPs - α, β, and αβ - and until now there have been no reports of human AMPs with a non-αβ structure (Wang, 2014). The most studied AMPs in humans, i.e., cathelicidin (LL-37), α-defensins and β-defensins, are cationic and belong to the α-helical, β-sheet, and αβ peptides respectively. Bovine indolicidin, a 13-residue cationic AMP rich in tryptophan and proline, has been the model peptide representing the non-αβ class for almost two decades since its NMR structure was determined (Rozek et al., 2000). In the current study, we have identified a non-αβ class of human AMPs, in which KAMP-19 and its proteolytic variants do not contain regular α-helical structures as observed in LL-37 or regular β-sheet structures as observed in human α-defensins. KAMP-19, a glycine-rich peptide without any tryptophan is distinct from the tryptophan-rich indolicidin peptide. This non-αβ NMR structure of KAMPs is consistent with CD spectra, which do not suggest any regular α-helical or β-sheet structure (Figures 1, 2). However, there are 3_10_ helical turns and bends in the structure that allow the KAMP peptide chain to fold into a membrane-binding scaffold. The hydrophobic strip (Figure 3) of the peptide binds to the *P. aeruginosa* inner and outer membranes, and is therefore a unique addition to the known 3D structures of human AMPs.

The mechanism of action of cationic helical/β-sheet AMPs demonstrated that their initial binding to anionic bacterial membrane is heavily dependent on electrostatic attractions, and that their membrane perturbation activity requires defined secondary structures in the bacterial membrane environment. For example, basic Arg23 and hydrophobic phenylalanines of the LL-37 major antimicrobial peptide directly interact with anionic lipid phosphatidylglycerols, which are essential for perturbation of the bacterial cell membrane (Wang, 2007; Wang et al., 2014). However, the mechanism by which non-cationic, non-αβ AMPs approach, bind and interact with bacterial membrane remains unclear. Furthermore, many Gram-positive and -negative bacteria have developed mechanisms to resist cationic AMPs through reducing negative charge on the cell surface and modifying membrane lipid composition (Nawrocki et al., 2014; Band and Weiss, 2015). Non-cationic AMPs with low structural requirement (high structural flexibility) for bactericidal activity likely work together with cationic AMPs to generate an effective innate defense system. These atypical AMPs and their derivatives, including KAMPs described here, represent an underexplored group of peptide-based anti-infective candidates that are worth further investigation.

Specific interaction with the highly abundant, negatively charged bacterial surface components, LPS and teichoic acids in particular, is a key step for many cationic AMPs such as LL-37 (Wang, 2008) prior to their subsequent actions to disrupt or translocate across the membrane. As a result, bacterial mutants with deficient, truncated or modified LPS or teichoic acids can become resistant to these AMPs (Wang et al., 2014). For example, Gram-negative *Acinetobacter baumannii* with adaptive mutations in LPS biosynthesis genes (*lpxA, lpxC, lpxD*) or which confer structural modifications of LPS lipid A (*pmrA, pmrB*) are highly resistant to colistin, the cationic peptide antibiotic that is the last resort treatment for multi-drug resistant bacteria (Moffatt et al., 2010; Park et al., 2015). Also, *Escherichia coli* and *Salmonella typhimurium* with *waaY* gene mutation that affects heptose II phosphorylation of the LPS inner core have decreased susceptibility to LL-37 (Lofton et al., 2013; Bociek et al., 2015); D-alanylation of LTA catalyzed by the *dlt* operon enables Group B *Streptococcus* resistance to cationic AMPs including LL-37, magainin 2 and colistin (Saar-Dover et al., 2012). In marked contrast, KAMP-10 and KAMP-19 interactions with any of the three LPS components (O-antigen, core and lipid A) is not required for bactericidal activity, suggesting that KAMPs might have an advantage over the above classic cationic AMPs to overcome this common resistance mechanism and remain effective against LPS mutants, and that those KAMPs might be less likely to induce bacterial resistance that involves LPS modification. However, as lipid A is known to activate TLR4-mediated pro-inflammatory signaling cascade, the low binding affinity of KAMP-10 and KAMP-19 for lipid A would be expected to weaken their ability to neutralize LPS specifically through the direct binding mode. Nevertheless, other mechanisms for AMPs to modulate LPS-mediated inflammatory responses exist, including competitive inhibition via TLR4/MD-2/CD14 receptor binding (Nagaoka et al., 2001), inhibition of TLR4 endocytosis and TRIF-dependent signaling (Shim et al., 2015), and perhaps direct induction of anti-inflammatory gene expression through other receptors. On the other hand, the relatively high binding affinity of KAMP-18C and KAMP-36 for LTA, the Gram-positive cell wall component that has endotoxin properties similar to those of LPS, would likely correlate with their ability to block LTA-induced inflammatory responses through direct neutralization, as demonstrated previously for some AMPs (Scott et al., 1999). Further studies are required to characterize the interaction of KAMPs with LPS and LTA.

Increasing evidence has shown that binding to specific cell surface receptors can facilitate the antimicrobial activity of AMPs. For instance, hRNase7 binds to *P. aeruginosa* OprI (outer membrane protein I) (Lin et al., 2010), and LL-37 to Enterobacteriaceae Lpp (a major outer membrane lipoprotein) (Chang et al., 2012) and *A. baumannii* OmpA (outer membrane protein A) (Lin et al., 2015). In the current study, we identified a potential *P. aeruginosa* surface receptor for KAMP-19. The hypothetical protein PSPA7_4325 of *P. aeruginosa* strain 6206 is 99.4% homologous to the outer membrane lipoprotein SlyB of another clinical isolate DK1. SlyB is well conserved in Gram-negative bacteria and contributes to the integrity of cell envelope (Plesa et al., 2006). Interestingly, expression of SlyB can be upregulated by the PhoP-PhoQ system that is activated under stressful conditions such as low magnesium (Perez et al., 2009), low pH (Prost et al., 2007), or the presence of cationic AMPs (Bader et al., 2005), i.e., those with high positive charge, hydrophobicity and amphipathicity (Shprung et al., 2012). The bacterial two-component system is also known to enable various resistance mechanisms against cationic AMPs. If SlyB is shown to facilitate the bactericidal activity of KAMPs, it would be of interest to investigate further whether KAMPs are effective under the conditions that are unfavorable to cationic AMPs, and to examine if there is any synergism between KAMPs and other cationic AMPs due to their activation of PhoPQ.

Using electron microscopy, we observed that KAMP-10 formed pores at the inner and outer membranes of *P. aeruginosa*. The NMR structures of KAMP-19 and KAMP-10 provide a basis for such interactions of the peptides with membranes. Owing to the plasticity of the glycine strings, we speculate that such a peptide structure may work equally well for KAMPs to associate with various molecular partners. Our work suggests that NqrA and the ribosomal machinery, among others, are potential intracellular targets of KAMPs. NqrA is one of the six subunits of Na^+^-NQR cytoplasmic membrane complex responsible for generating the transmembrane electrochemical gradient of Na^+^ critical for virulence and survival of many pathogenic bacteria. The bioenergetic sodium pump is not found in eukaryotes and has been recognized as a potential target for new antibiotic development (Dibrov, 2013). Structural characterization of Na^+^-NQR complex revealed that NqrA is tightly bound to the cytoplasmic side of NqrB that harbors the Na^+^ channel (Steuber et al., 2015); therefore targeting NqrA might block the channel entrance or alter the conformation of NqrB to block ion channel activity. It is possible that KAMPs employ multiple antimicrobial strategies from disrupting cell envelopes to inhibiting cellular respiration and/or biosynthesis, which can make it more difficult for bacteria to become resistant.

In conclusion, KAMP-19 and its proteolytic variants are the first non-αβ antimicrobial peptides reported in humans. These glycine-rich AMPs derived from epithelial keratin 6A have a new amphipathic structure; the hydrophobic strip including glycine residues is important for peptide-bacterial membrane interactions. We show that these salt-tolerant peptides do not require LPS and LTA binding for their initial access to bacterial membranes; lipoprotein SlyB, highly conserved in Gram-negative bacteria, might be the preferred docking molecule on the outer membrane. We also demonstrate that KAMPs can damage bacterial envelopes, and potentially target intracellular machinery such as the respiratory sodium pump (Na^+^-NQR) and the ribosomes. Further studies will be required to advance our understanding of the modes of bactericidal action and to determine if there are additional functions for KAMPs. While the growth of knowledge about natural AMPs and the development of synthetic anti-infective peptides are fueled by the studies on canonical helices, this study highlights the first member for a non-canonical class of human AMPs and may inspire new designs of peptide-based antibiotics.

## Author contributions

JL: Performed and coordinated all experiments; YT: Performed Zetasizer measurements; GW: Performed NMR experiments and determined the structure; JL, GW, and CT: Analyzed the data; CT: Conceived and directed the project; CT, GW, and JL: wrote the manuscript.

## Funding

This study was supported by research funds from Cleveland Clinic, Cleveland, OH (CT) and grants R01 EY023000 from the National Eye Institute (CT) and R01 AI105147 from the National Institute of Allergy and Infectious Diseases (GW) of NIH.

### Conflict of interest statement

JL, GW, and YT declare that the research was conducted in the absence of any commercial or financial relationships that could be construed as a potential conflict of interest. CT is listed as a co-inventor on US Patent issued November 17, 2015, No. 9,187,541 B2, entitled “Anti-Microbial Peptides and Methods of Use Thereof.”
